# P05.02. Constructing an integrative medicine care service in a Brazilian University hospital

**DOI:** 10.1186/1472-6882-12-S1-P362

**Published:** 2012-06-12

**Authors:** P Siegel, N Filice de Barros, J Carvalheira

**Affiliations:** 1 State University of Campinas, Campinas, Brazil

## Purpose

The purpose of this paper is to highlight the partial results of the first project on Integrative Oncology in Brazil, which is being put into practice at the Clinics Hospital of the University of Campinas (HC/Unicamp). The majority of the Brazilian population uses the public National Health System (SUS) and one of its units, the National Institute for Cancer (INCA), administers cancer treatments. Although in 2006 the National Policy for Complementary and Integrative Practices (PNPIC) was established allowing for homeopathy, acupuncture, medicinal herbs, Thermalism and Anthroposophical Medicine to be incorporated into the SUS, there is no mention yet of Integrative Oncology at the INCA.

## Methods

The project is designed with qualitative methodology and thirty-two members of the cancer unit staff were interviewed on what they think about applying integrative and complementary practices (ICP) to cancer patients and which ICPs they recommend.

## Results

The totality, except one, of the staff members was in favor of applying ICPs to cancer patients, yet the reasons they offered were grouped together into four different categories. In the first one, staff members explained that patients need wanted types of care other than chemo and radiotherapy; the second category referred to the specific use of ICPs to treat oncological pain; the third category emphasized well-being and feeling cared for, and the last category dealt with the possibility of applying ICPs outside the hospital, meaning patients would be referred to some other service. Acupuncture, reiki, medicinal herbs and yoga were the most frequently suggested ICP with the aim of alleviating pain and enhancing well-being.

## Conclusion

The project intends to launch the discussion on Integrative Oncology in the Brazilian National Health System.

